# Myasthenia gravis following human papillomavirus vaccination: a case report

**DOI:** 10.1186/s12883-018-1233-y

**Published:** 2018-12-28

**Authors:** Ji Yeon Chung, Seung Jae Lee, Byoung-Soo Shin, Hyun Goo Kang

**Affiliations:** 1 Department of Neurology, Chosun University School of Medicine, Gwangju, 61453 Republic of Korea; 2 Institute for Molecular Biology and Genetics and Department of Chemistry, Chonbuk National University, Jeonju, 54896 Republic of Korea; 3 Department of Neurology, Chonbuk National University School of Medicine, Jeonju, 54896 Republic of Korea; 4 Department of Neurology & Research Institute of Clinical Medicine of Chonbuk National University - Biomedical Research Institute of Chonbuk National University Hospital, 20 Geonji-ro, Deokjin-gu, Jeonju-si, Jeonbuk-do 54907 South Korea

**Keywords:** Adverse event, Human papillomavirus vaccine, Myasthenia gravis, Myasthenia gravis crisis, Vaccination

## Abstract

**Background:**

Myasthenia gravis (MG), an autoimmune neuromuscular disorder, occurs owing to autoantibodies against acetylcholine receptors. MG symptoms can be triggered by various vaccines. Many studies have evaluated the safety and adverse events of the human papillomavirus (HPV) vaccine. Here, we present a life-threatening case of ocular and bulbar MG symptoms after HPV vaccination and a brief literature review.

**Case presentation:**

A 23-year-old woman presented with binocular diplopia, ptosis, dysarthria, and dysphagia, which occurred on the 3rd day after the second HPV vaccine administration. She was diagnosed with MG based on history, clinical features, and test results. Her symptoms deteriorated on the 3rd day after admission, and she was transferred to the intensive care unit with mechanical ventilation. On the 7th day after admission, due to discomfort in the right chest, pulmonary embolism was suspected. A tracheostomy was performed on the 14th day of mechanical ventilation. In the 4th week, the tracheostomy tube was removed; all symptoms had completely resolved at discharge. She was followed up for 5 months without recurrence or further treatment.

**Conclusion:**

HPV vaccination may cause MG owing to unexpected abnormal autoimmune responses. Additional studies are needed to clarify the possible causal relationship between the HPV vaccine and neurological complications and to evaluate the safety of the vaccine.

## Background

Myasthenia gravis (MG) is an autoimmune disease that causes a neuromuscular junction disorder owing to blockage of the nicotinic acetylcholine receptor (AChR). MG may be associated with autoimmune reactions, such as autoantibodies and autoimmune responses against AChR [1]. Furthermore, MG is related to thymus disorders and other autoimmune diseases [2]. The human papillomavirus (HPV) vaccination was developed to prevent cervical cancer and is recommended for female individuals aged 9–26 years. In South Korea, bi- and quad-rivalent vaccines have been used; however, since 2016, the nine-valent vaccine has been used, with no serious adverse effects reported to date. Here, we present a case of MG after HPV nine-valent vaccination in a patient whose condition rapidly progressed to MG.

### Case presentation

A 23-year-old woman with binocular vertical diplopia, bilateral ptosis (which worsened with left and down gazing), dysarthria, and dysphagia visited the outpatient department. She had received a primary HPV nine-valent vaccination 2 months prior and a second vaccination 5 days before the visit. The symptoms occurred on the 3rd day after the second vaccination. The muscular strength of her upper and lower extremities was normal, and the deep tendon reflex of both sides was normal. Her ptosis and diplopia temporarily improved with an ice pack and pyridostigmine test. The repetitive nerve stimulation (RNS) did not reveal a significant decrement in deltoid, abductor digiti minimi, flexor carpi, and orbicularis oculi muscles. The serum AChR antibody titer was 1.66 nmol/L. Other autoimmune disease tests, including rheumatoid factor and antinuclear antibody, were negative. A thyroid function test was normal, and no thymus abnormality was observed on chest computed tomography (CT).

**Fig. 1:**
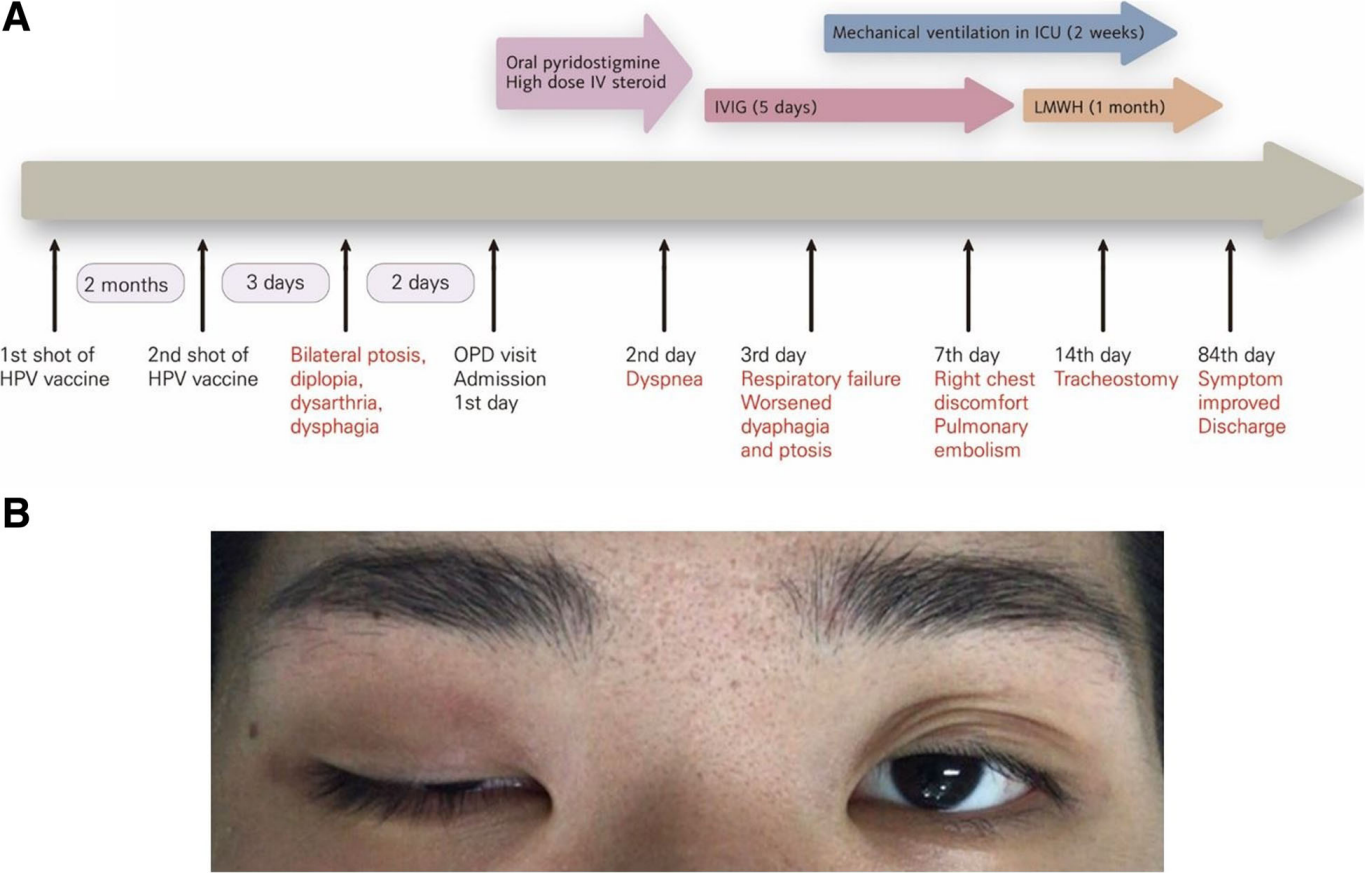
**a** Timeline of the patient. **b** A 23-year-old woman with anti-acetylcholine receptor-positive myasthenia gravis. Right upper eyelid ptosis worsened to almost complete closure, and respiratory muscle weakness resulted in intubation and mechanical ventilation on hospital day 3

She was diagnosed with MG, and pyridostigmine oral administration and high-dose intravenous steroid therapy were initiated. Her dyspnea became more severe on the 2nd day after admission, and oxygen saturation decreased; therefore, she received intravenous immunoglobulin therapy. Afterward, spontaneous breathing became more difficult, and the dysphagia and bilateral ptosis worsened. These symptoms were determined to demonstrate an MG crisis, and mechanical ventilation was initiated after endotracheal intubation (Fig. 1). Although muscle strength was normal at admission, the extension power of the distal fingers decreased.

On the 7th day after admission, sudden tachycardia was observed, the patient experienced persistent pressure in the right chest, and oxygen saturation decreased during mechanical ventilation. Chest CT revealed a low-density-filling defect of the pulmonary artery in the right lower lobe. Ischemic changes in the lung parenchyma and an increased D-dimer level (2199 ng/mL) were also observed. Therefore, heparin treatment was initiated owing to the possibility of pulmonary embolism. The D-dimer level decreased to within the normal range (99 ng/mL) in the 2nd week after the increase.

In the 4th week of hospitalization, the symptoms further improved such that she could walk and the AChR antibody titer decreased (0.99 nmol/L). However, right eye ptosis and binocular vertical diplopia persisted. She was discharged after the dysphagia had completely resolved. The AchR antibody titer was normal (0.05 nmol/L) at discharge. The patient has returned to daily life without symptom recurrence or further treatment.

## Discussion

The HPV vaccination was first approved in 2006 for preventing cervical cancer. However, in Japan, serious adverse events, such as Guillain-Barré syndrome, acute disseminated encephalomyelitis, postural orthostatic tachycardia syndrome, and complex regional pain syndrome have been reported in patients receiving the HPV vaccination, which were suspected to be associated with the HPV vaccination [3]. The causal relationship between these adverse events and HPV vaccination has not yet been elucidated, and the underlying pathogenesis remains unclear. Studies in Japan have hypothesized that an antibody that cross-reacts with autonomic ganglia, neurons, and cardiac proteins or ß1/2-adrenergic and M2/3 muscarinic receptors could be synthesized owing to the epitope of the HPV vaccination [4], and that cytotoxic T cells could be activated by stimulating the antibody production by binding to acetylcholine receptors [5]. A recent study reported a complex involving HPV and p53 pro-apoptotic tumor suppressor, and the inhibitory enzyme is degraded upon complex formation [6]. Viral oncoprotein E6 can recognize a short leucine-rich consensus sequence within ligase E6AP, and this complex finally degrades p53. The E6 domain has two zinc ions, which maintain structural features for the interactions. Non-specific interactions of HPV with AChR may result in the complex formation and unexpected side effects of HPV, which need to be investigated.

Our patient had no problems with the primary HPV vaccination but exhibited acute bilateral ptosis, dysarthria, and dysphagia on the 3rd day after the second vaccination. She also experienced acute respiratory failure and pulmonary embolism. In our case, blood stasis owing to immobilization while in the intensive care unit could be a risk factor for pulmonary embolism. However, because venous thromboembolism was reported as an adverse event of the HPV vaccination [7], pulmonary embolism could occur owing to the HPV vaccination.

In our patient, MG may have been induced by the HPV vaccination as an adverse event or incidentally without an association between the two factors. Therefore, it may be difficult to suggest a strong relationship between HPV vaccination and MG outbreak. However, previous studies have reported MG occurrence after inoculation with other vaccines [8–10], suggesting an association between symptoms and changes in immune responses in the body following vaccination. This study summarized the studies that reported the first occurrence of MG after vaccinations based on the available literature (Table 1). Additionally, there is no absolute contraindication to the use of the HPV vaccination. Our patient developed MG after receiving the nine-valent vaccine, and the relative risk of the nine-valent vaccine is unclear.

**Table 1:** Case reports of new-onset myasthenia gravis after vaccination

Author	Age/Sex	Vaccine Type	Time to onset	Initial symptoms	Treatment	Prognosis (Time to recovery)	Thymoma	MGFA Class
Biron [9]	48/M	HBV	1 mo after 2nd shot	Ocular	Edrophonium,, plasma exchange, cyclophosphamide, steroid	Improved. (After 30 PE)	+	I
Bahri [10]	46/F	HBV	1mo after 2nd shot	Ocular, bulbar	Pyridostigmine, steroid	Improved (Not mentioned)	+	IIb
Takizawa [8]	69/M	BCG	6 wks	Ocular	Pyridostigmine	Improved (70 days)	–	IIa
Our case	23/F	HPV	3 d after 2nd shot	Ocular, bulbar	Pyridostigmine, steroid, IVIG	Improved (84 days)	–	V

*Mo* months, *d* days, *HBV* hepatitis B vaccine, *BCG* Bacillus Calmette-Guerin, *HPV* human papillomavirus, *IVIG* intravenous immunoglobulin, *MGFA* myasthenia gravis foundation of America clinical classification

This case report implies that the HPV vaccination may cause MG. Other neurological manifestations may occur owing to unexpected abnormal autoimmune responses such as autonomic dysfunction and pain. It is important to inform patients prior to inoculation and observe the occurrence of abnormal symptoms. Moreover, it is critical to intervene promptly and treat the patient when fatal deterioration is observed. We believe that additional studies are needed to assess the possible causal relationship between the HPV vaccine and neurological complications and to evaluate the safety of the vaccine.

## Abbreviations

AChR: Acetylcholine receptor
CT: Computed tomography
HPV: Human papillomavirus
MG: Myasthenia gravis
